# Identification and implications of a core bacterial microbiome in 19 clonal cultures laboratory-reared for months to years of the cosmopolitan dinoflagellate *Karlodinium veneficum*

**DOI:** 10.3389/fmicb.2022.967610

**Published:** 2022-08-04

**Authors:** Yunyan Deng, Kui Wang, Zhangxi Hu, Qiang Hu, Ying Zhong Tang

**Affiliations:** ^1^CAS Key Laboratory of Marine Ecology and Environmental Sciences, Institute of Oceanology, Chinese Academy of Sciences, Qingdao, China; ^2^Laboratory of Marine Ecology and Environmental Science, Qingdao National Laboratory for Marine Science and Technology, Qingdao, China; ^3^Center for Ocean Mega-Science, Chinese Academy of Sciences, Qingdao, China; ^4^Institute for Advanced Study, Shenzhen University, Shenzhen, China; ^5^College of Fisheries, Guangdong Ocean University, Zhanjiang, China; ^6^Faculty of Synthetic Biology, CAS Key Laboratory of Quantitative Engineering Biology, Shenzhen Institute of Synthetic Biology, Shenzhen Institute of Advanced Technology, Chinese Academy of Sciences, Shenzhen, China

**Keywords:** *Alcanivorax*, core bacterial microbiome, hydrocarbon-degrading bacteria, *Karlodinium veneficum*, *Ponticoccus*, *Thalassospira*

## Abstract

Identification of a core microbiome (a group of taxa commonly present and consistently abundant in most samples of host populations) is important to capture the key microbes closely associated with a host population, as this process may potentially contribute to further revealing their spatial distribution, temporal stability, ecological influence, and even impacts on their host’s functions and fitness. The naked dinoflagellate *Karlodinium veneficum* is a cosmopolitan and toxic species, which is also notorious in forming harmful algal blooms (HABs) and causing massive fish-kills. Here we reported the core microbiome tightly associated with 19 strains of *K. veneficum* that were originally isolated from 6 geographic locations along the coast of China and from an estuary of Chesapeake Bay, United States, and have been maintained in the laboratory for several months to over 14 years. Using high-throughput metabarcoding of the partial 16S rRNA gene amplicons, a total of 1,417 prokaryotic features were detected in the entire bacterial microbiome, which were assigned to 17 phyla, 35 classes, 90 orders, 273 families, and 716 genera. Although the bacterial communities associated with *K. veneficum* cultures displayed heterogeneity in feature (sequences clustered at 100% sequence similarity) composition among strains, a core set of 6 genera were found persistent in their phycospheres, which could contribute up to 74.54% of the whole bacterial microbiome. Three γ-proteobacteria members of the “core,” namely, *Alteromonas*, *Marinobacter*, and *Methylophaga*, were the predominant core genera and made up 83.25% of the core bacterial microbiome. The other 3 core genera, *Alcanivorax*, *Thalassospira*, and *Ponticoccus*, are reported to preferably utilize hydrocarbons as sole or major source of carbon and energy, and two of which (*Alcanivorax* and *Ponticoccus*) are recognized as obligate hydrocarbonoclastic bacteria (OHCB). Since OHCB generally present in extremely low abundance in marine water and elevate their abundance mostly in petroleum-impacted water, our detection in *K. veneficum* cultures suggests that the occurrence of obligate and generalist hydrocarbon-degrading bacteria living with dinoflagellates may be more frequent in nature. Our work identified a core microbiome with stable association with the harmful alga *K. veneficum* and opened a window for further characterization of the physiological mechanisms and ecological implications for the dinoflagellate-bacteria association.

## Introduction

In aquatic habitats, phytoplankton communities within the photic zones are characterized by highly heterogeneous assemblages of unicellular microalgae and a wide variety of associated microbes. The phototrophic microalgae capture solar energy, transform inorganic matter into organic matter, and release organic compounds to the region immediately surrounding themselves, known as the phycosphere, which is enriched in organic molecules exuded by the algal cell into the surrounding water, and is the aquatic analogue of the rhizosphere (Seymour et al., 2017; Durán et al., 2022). The phycosphere provides a niche for colonization of heterotrophic bacteria, which utilize a major fraction of fixed carbon through catabolism of microalgal metabolites. It could be considered as a marketplace where cross-kingdom communications are mediated by the release and uptake of organic compounds (Kouzuma and Watanabe, 2015; Seymour et al., 2017). The exchange of metabolites and infochemicals at this interface governs multifarious and sophisticated microalgae-bacteria associations, which span from cooperative to competitive relationships (e.g., mutualism, commensalism, parasitism and competition; Amin et al., 2009; Kouzuma and Watanabe, 2015; Aharonovich and Sher, 2016; Hennon et al., 2018). Since modulating nutrient cycling and biomass production at the base of the marine food web, interactions between microalgae and bacteria represent one of the fundamental ecological relationships in aquatic environments, which are considered to have multilevel influences and substantial impacts on ecosystem-scale processes (e. g. biogeochemical cycling, ecosystem productivity, harmful algal blooms; Buchan et al., 2014; Seymour et al., 2017).

The concept of “core microbiome” originally emerged for human microbiome and refers to common groups of microbes or genes that were likely to be particularly important for host biological function (Shade and Handelsman, 2012 and the references therein), and further expanded to other host-associated microbiomes. Broadly, the core microbiome has been referred to any set of microbial taxa, or the genomic and functional attributes associated with those taxa, that are characteristic of a host or environment of interest (Astudillo-Garcia et al., 2017; Risely, 2020; Neu et al., 2021). Most commonly, core microbiomes are measured as the suite of taxa shared among two or more samples from a particular host or environment (Shade and Handelsman, 2012; Astudillo-Garcia et al., 2017; Neu et al., 2021). Identifying the core members is the first step to understand the stable, consistent components across complex microbial assemblages, because those commonly occurring taxa that appear in all assemblages are proposed to be associated with a particular habitat and are critical to the function of that type of community (Shade and Handelsman, 2012; Astudillo-Garcia et al., 2017; Risely, 2020; Neu et al., 2021). For terrestrial plants, the composition and function of core microbiomes within a rhizosphere or phyllosphere have been explored in some model plants, such as *Arabidopsis*, maize, rice, barley, and soybean (Lundberg et al., 2012; Peiffer et al., 2013; Mendes et al., 2014; Edwards et al., 2015). Several works conveyed a notion that the soil types and host plant genotypes were the main factors affecting the associated bacterial consortia (Lundberg et al., 2012; Peiffer et al., 2013; Edwards et al., 2015; Grady et al., 2019), though a few of them addressed the effects of microbial assemblages on host metabolism (Risely, 2020 and the references therein).

Dinoflagellates are the second most abundant phytoplankton in coastal marine ecosystems and infamous for causing harmful algal blooms (HABs). Approximately 200 species in this lineage have been recorded as HABs-causing agents, accounting for ca. 40% of the total recorded species forming HABs globally (Jeong et al., 2021). With the ever-increasing number of dinoflagellate HAB events worldwide in the past decades, the importance of dinoflagellate-bacteria interactions have been increasingly emphasized due to amassed evidence of linkages between bacterioplankton and the population dynamics of dinoflagellates in the blooming field (Mayali et al., 2011; Yang et al., 2015; Zhou et al., 2018; Hattenrath-Lehmann et al., 2019; Cui et al., 2020; Kang et al., 2021; Maire et al., 2021). Meanwhile, bacteria within certain lineages (e.g., *Roseobacter* clade in α-proteobacteria, *Marinobacter* and *Alteromonas* clades in γ-proteobacteria) have been commonly found both in the phycospheres of laboratory-cultured dinoflagellates (Bolch et al., 2017; Park et al., 2018; Shin et al., 2018; Li et al., 2019; Guo et al., 2020; Tarazona-Janampa et al., 2020; Deng et al., 2022; Wu et al., 2022) and field samples during dinoflagellate blooms (Mayali et al., 2011; Yang et al., 2015; Zhou et al., 2018; Hattenrath-Lehmann et al., 2019; Cui et al., 2020; Kang et al., 2021), implying that specific interactions could be established between dinoflagellates and particular groups of bacteria. However, the relationships between phytoplankton and surrounding bacteria are intimate, complex, and dynamic (Amin et al., 2012; Behringer et al., 2018; Tarazona-Janampa et al., 2020; Maire et al., 2021; Wu et al., 2022). Compared with microbiota of sessile plants, the bacterial consortia co-existed with phytoplankton in aquatic habitats are particularly difficult to be accurately defined, owing to the majority of microalgae being unicellular organisms living in constantly changing and dynamic environments (Behringer et al., 2018). Furthermore, some case studies recorded that the community composition of the associated bacterial flora was correlated with the growth stage of host phytoplankton (Grossart et al., 2005; Lupette et al., 2016; Bolch et al., 2017; Park et al., 2018; Li et al., 2019), suggesting that their interactions are more complex than expected. Although previous works have highlighted some common and/or important bacterial taxa interacting with many dinoflagellate species, the association of specific bacterial taxa with multiple strains of a specific dinoflagellate species, which would suggest “intimate” associations between the hosting species and its “microbiome” (Behringer et al., 2018), has become a new research perspective and hotspot. Recent research efforts have been undertaken to investigate Symbiodiniaceae members, the crucial endosymbionts of coral reefs (Lawson et al., 2018; Maire et al., 2021). Lawson et al. (2018) characterized the bacterial communities association with a wide phylogenetic diversity of *Symbiodinium* cultures (18 types spanning 5 clades), showing the existence of a consistent core bacterial microbiome among different *Symbiodinium* clades. From 11 cultures of Symbiodiniaceae covering 6 genera and 9 species, Maire et al. (2021) identified a highly conserved suite of intracellular bacteria across all these species of Symbiodiniaceae, suggesting these bacteria are involved in Symbiodiniaceae physiology. The area of core bacterial microbiome associated with dinoflagellates is still in the mist and underexplored.

The naked *Karlodinium veneficum* (Ballantine) J. Larsen is a notorious harmful algal bloom-forming dinoflagellate, which has a cosmopolitan distribution covering coastal ecosystems of all continents except Antarctica (Yang et al., 2020 and the references therein). Since first been isolated form United Kingdom, numerous bloom events of *K. veneficum* have been reported worldwide, e.g., South Africa, Europe, North America and Australia (as reviewed in Yang et al., 2019). In China, *K. veneficum* blooms have increasingly occurred since 2000. *K. veneficum* is a toxic species that produces karlotoxins, which have lethal effect to predators (Deeds et al., 2002) and may also lead to human skin irritation, swelling, rashes and itchiness (Lim et al., 2014). In addition, the species is an omnivorous phagotroph that could obtain nutrients and energy via feeding on aquatic organisms (Yang et al., 2020), and also exhibits potent allelopathic effects on other co-occurring algae (Yang et al., 2019). Due to these characteristics, its blooms usually cause massive mortality of fishes, mussels and zooplankton (as reviewed in Yang et al., 2021). For instance, a long-lasting *K. veneficum* bloom along the Yellow Sea coastal waters (Sanggou Bay, Shandong Province) in 2012 caused severe impacts on the local aquaculture (Xu et al., 2012). Massive fish-kills caused by *K. veneficum* were frequently reported in the Chesapeake Bay, United States from 2015 to 2017, killing nearly half million fish (Maryland Department of the Environment, 2017). *K. veneficum* was also responsible for the fish kill in coastal Finnish waters (Ersöströmmen, Finland) in 2016, which is the first case shown to be related to this species in the Baltic Sea (Finnish Environment Institute, 2016). Given the frequent bloom events of *K. veneficum* and their severe impacts on fishery and aquaculture, it has drawn great attention from the scientific community and thus has been intensively studied from multiple aspects (toxicology, toxins, trophic mode, blooming dynamics, allelopathy, and life cycle). As an important facet of its ecology, the bacterial consortia harbored by this species, however, have not been adequately examined. During the past years, we have established 19 clonal cultures *K. veneficum* that have been isolated from 6 geographic regions along the coast of China and from an estuary of Chesapeake Bay, United States, and maintained for months to more than 14 years, which provided us a highly valuable asset in terms of the abovementioned research objective. Therefore, we characterized the bacterial communities associated with these laboratory cultures of *K. veneficum* with the aim to define the core (constitutive) bacterial microbiome that stably co-exists with the species. Our findings provided new insights into the possible mechanisms underpinning the possible intimate associations and their potential contributions to physiological functioning of *K. veneficum.*

## Materials and methods

### Algal cultures of *Karlodinium veneficum*

A total of 19 strains of *K. veneficum* were employed in this study. The strain KV7 + 8, was isolated from the Chesapeake Bay estuary, United States in 2006 (Table 1 and Supplementary Figure 1). The other 18 strains were isolated from 5 different geographic origins of China (Fujian, Guangdong, Guangxi, Hebei, and Shandong Provinces; Table 1 and Supplementary Figure 1), which were initially established via single-cell pipetting either from coastal waters or via resting cyst germination from 2014 to 2020 (see Table 1 for details). The samples harvested for genomic DNA isolation (see below) were performed on December 20, 2020, thus these cultures have been maintained in the laboratory for several months to over 14 years. All cultures were routinely maintained in f/2-Si medium (i.e., f/2 medium without NaSiO_3_; Guillard, 1975) prepared with sterilized and filtered (0.22 mm; Millipore Corp.) seawater (salinity 32–33), in an incubator that was set at a temperature of 20 ± 1°C, an light/dark cycle of 12:12 h L/D, and an irradiance intensity of 100 μmol photons m^–2^ s^–1^.

**TABLE 1:** Information on 19 clonal cultures of *Karlodinium veneficum* used in this study.

Sample ID	Strain number	Origin	Date of isolation
KV1	KVND-1-cyst	Ningde (26.88 °N, 120.19 °E), Fujian, China	Cyst germination, 2016
KV2	KVND1	Ningde (26.88 °N, 120.19 °E), Fujian, China	Vegetative cell, 2016
KV3	KVBDH1	Beidai Estuary (39.80 °N, 119.45 °E), Hebei, China	Vegetative cell, 2014
KV4	KV7 + 8	Chesapeake Bay (36.89 °N, 76.33 °W), Virginia, United States	Vegetative cell, 2006
KV5	KVJZBXG02	Jiaozhou Bay (36.08 °N, 120.32 °E), Shandong, China	Vegetative cell, 2019
KV6	KVJZBXG01	Jiaozhou Bay (36.08 °N, 120.32 °E), Shandong, China	Vegetative cell, 2019
KV7	DYKV7	Dongying (37.53 °N, 118.97 °E), Shandong, China	Vegetative cell, 2020
KV8	KVJZBXG2020-8	Jiaozhou Bay (36.08 °N, 120.32 °E), Shandong, China	Vegetative cell, 2020
KV9	I-K1-KV	Ningde (26.88 °N, 120.19 °E), Fujian, China	Vegetative cell, 2019
KV10	KVJZBXG04	Jiaozhou Bay (36.08 °N, 120.32 °E), Shandong, China	Vegetative cell, 2019
KV11	KVJZBXG05	Jiaozhou Bay (36.08 °N, 120.32 °E), Shandong, China	Vegetative cell, 2019
KV12	KVJZBXG06	Jiaozhou Bay (36.08 °N, 120.32 °E), Shandong, China	Vegetative cell, 2019
KV13	KVJZBXG07	Jiaozhou Bay (36.08 °N, 120.32 °E), Shandong, China	Vegetative cell, 2019
KV14	KVJZBXG08	Jiaozhou Bay (36.08 °N, 120.32 °E), Shandong, China	Vegetative cell, 2019
KV15	KVJZBXG09	Jiaozhou Bay (36.08 °N, 120.32 °E), Shandong, China	Vegetative cell, 2019
KV16	KVPRE1	Pearl River Estuary (22.28 °N, 113.58 °E), Guangdong, China	Vegetative cell, 2019
KV17	P4-1	Beibu Gulf (20.50 °N, 108.62 °E), Guangxi, China	Vegetative cell, 2018
KV18	P4-2	Beibu Gulf (20.17 °N, 108.61 °E), Guangxi, China	Vegetative cell, 2018
KV19	P4-3	Beibu Gulf (21.17 °N, 109.60 °E), Guangxi, China	Vegetative cell, 2018

### Sample collection and genomic deoxyribonucleic acid extraction

Cultures at exponential growth stage were inoculated into six-well culture plates (Corning, United States) containing 10 mL of the seawater-based f/2-Si medium and then incubated for 15 days under the same temperature and illumination as the routine culture conditions. Samples were then collected when all cultures were at their stationary growth stage as pre-determined. All cells in each sample (approximately 10^4^∼10^5^ cells) were pelleted in a 1.5 mL centrifuge tube and immediately used for genomic DNA isolation. Genomic DNA was extracted using the Plant DNA Extraction Kit (Tiangen, Beijing, China) referring to the manufacturer’s protocols. The total DNA of each sample was eluted with 50 μl TE buffer. Nuclear-free water processed through DNA extraction were used as sample blanks. The DNA quality and purity were estimated by NanoDrop™ 1000 spectrophotometer (Thermo Fisher Scientific, United States), then stored at –80°C for further PCR amplification.

### 28S and 16S rRNA genes amplicon sequencing

The highly variable D2 domain and parts of the more conservative D1 and D3 domains of eukaryotic 28S rRNA gene was PCR amplified using an eukaryotic universal primer set of LSU335 (5’-ACCGATAGCA(G)AACAAGTA-3’) and LSU714 (5’-TCCTTGGTCCGTGTTTCA-3’), following the PCR protocol that described in Chai et al. (2018). The V3–V4 variable region of the bacterial 16S rRNA gene was amplified using the primer set of 341F (5’-CCTACGGGNGGCWGCAG-3’) and 805R (5’-GACTACHVGGGTATCTAATCC-3’; Logue et al., 2016). The PCR protocol were performed as that described in Deng et al. (2022). All PCR reactions were conducted in 25 μl volume including 12.5 μl of 2 × Phusion^®^ Hot Start Flex Master Mix, 2.5 μl of each primer (1 μM), and 50 ng of template DNA. Nuclease-free water served as negative control. The resulting amplicons were checked on an agarose gel electrophoresis and purified with the Gel Extraction Kit (Axygen Biosciences, United States). The size and quantity of the purified amplicon libraries were assessed on Agilent 2100 Bioanalyzer (Agilent, United States) and then pyrosequenced on the NovaSeq PE250 platform (LC-Bio Technology Company, Hangzhou, China).

### Sequencing data processing and bioinformatic analyses

The quality control of raw data was performed in Fqtrim software (version 0.9.7), and the chimeric reads were further excluded using Vsearch tool (version 2.0.3; Rognes et al., 2016). Generally, raw paired-end reads with 10 bp of minimal overlapping, the length less than 200 bp, average quality score less than 20, and 20% of maximum mismatch rate were discarded. Paired-end reads was sorted into samples based on their unique barcode and merged using FLASH (version 1.2.6; Magoè and Salzberg, 2011). The amplicon sequence variants (ASVs, sequences clustered at 100% sequence similarity) were generated with DADA2 package (version 3.6.1; Callahan et al., 2016). The eukaryotic and bacterial features aligned to NCBI GenBank and SILVA databases (retrieved on April 23, 2021), respectively, were denominated at the domain, phylum, class, order, family, and genus levels. Relative abundance of each feature was estimated based on its read counts normalized to the total number of good quality reads. Alpha diversity indices (Shannon diversity, Simpson evenness, Chao1 richness, Observed species richness, and Goods coverage) and beta diversity of PCoA (Principal coordinated analysis) based on the weighted-uniFrac distance were conducted with QIIME (version 2) plugin (Bolyen et al., 2019). Functional annotations of the presented common bacterial taxa were predicted from 16S rRNA gene-based microbial feature compositions using the PICRUSt algorithm (version 2.3.0-b) to make inferences from KEGG database (Douglas et al., 2020).

### Identification of the core bacterial microbiome of *Karlodinium veneficum* across different strains

In this study, bacterial taxa that were commonly and abundantly present across different samples were regarded as the core or stable constituents of the dinoflagellate-associated microbiome, a definition similarly adopted in the previous works on dinoflagellates (Lawson et al., 2018; Sörenson et al., 2019; Maire et al., 2021; Wu et al., 2022). Hence, the core microbiome was defined hereafter as those taxa present in 100% of all samples with a minimum relative abundance of 0.1%. The core bacterial microbiome was delineated by using the “get.coremicrobiome” function of the Mothur software platform with an abundance threshold of 0.1% (Astudillo-Garcia et al., 2017).

## Results

### General descriptions of pyrosequencing the eukaryotic 28S rRNA and prokaryotic 16S rRNA gene amplicons

The eukaryotic 28S rRNA gene pyrosequencing yielded 1,598,796 raw reads, with an average of 84,147 sequences per sample (Supplementary Table 1). The raw reads were deposited into the NCBI SRA database (BioProject: PRJNA824339). Good coverage value, an indicator of sample completeness (Chao and Jost, 2012), was 1.00 in all 19 samples (Supplementary Table 2), indicating that the sequencing depth sufficed to capture the feature diversity of eukaryotic taxa. Upon removing short sequences, poor quality sequences, and chimeric sequences, 1,546,779 clean sequences remained. After dereplication using DADA2 plugin within the QIIME 2 tool, the 28S rDNA sequencing data set finally generated 729 effective features (sequences clustered at 100% sequence similarity; Supplementary Table 3). Based on the annotations in NCBI database, features assigned to “*Karlodinium veneficum*” accounted for 97.13–99.93% of the total features in all 19 samples (Supplementary Table 3), manifesting the identity of all cultures used in our study as *K. veneficum*.

The prokaryotic 16S rRNA gene sequencing generated 1,601,282 raw reads (BioProject: PRJNA771505), with sequences per sample ranging from 80,045 to 87,348 (Supplementary Table 1). Goods coverage of all samples were 1.00 (Supplementary Table 2), indicating sufficient sequences were harvested to reveal the majority of taxa in the prokaryotic assemblages. A total of 1,375,395 clean reads were obtained from the 19 samples (Supplementary Table 1), with a quality control efficiency of 85.89%. The dereplication using DADA2 plugin yielded 1,458 effective features (Supplementary Table 3). Based on the assignments in Silva database, the 1,417 features were used for subsequent analyses, whereas the remained 41 features annotated as “unclassified” were excluded from further analysis.

### Feature composition of bacterial communities across strains of *Karlodinium veneficum*

**FIGURE 1:**
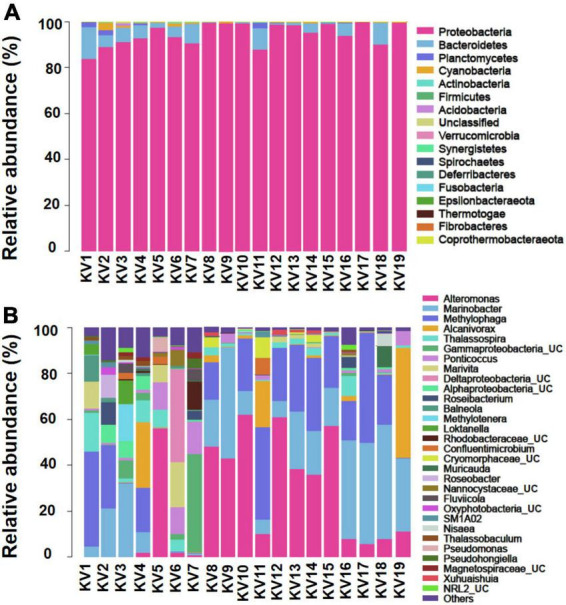
Relative abundance of bacteria at phylum **(A)** and genus [top 30; **(B)**] levels. The abundance is presented in terms of percentage in total effective features in a sample. UC, unclassified.

The 1,417 features identified as bacteria covered 17 phyla, 35 classes, 90 orders, 273 families, and 716 genera. The number of features per sample varied from 74 to 592 (mean = 157; Supplementary Table 3). Generally, Proteobacteria was the absolutely predominant phylum, account for 94.45% of all features, followed by Bacteroidetes (4.30%), Planctomycetes (0.47%), and Cyanobacteria (0.44%; Figure 1A). At the class level, the bacterial community was dominated by γ-proteobacteria (73.50%), α-proteobacteria (17.82%), δ-proteobacteria (3.03%), Bacteroidia (2.87%), and Rhodothermia (0.98%). At the genus level, *Alteromonas* (22.37%), *Marinobacter* (20.46%), *Methylophaga* (19.22%), *Alcanivorax* (5.74%), *Thalassospira* (3.52%), UC (unclassified) γ-proteobacteria (3.29%), *Ponticoccus* (3.22%), *Marivita* (2.48%), UC δ-proteobacteria (2.27%), UC α-proteobacteria (1.41%), and *Roseibacterium* (1.03%) were the most dominant genera for all samples as a whole (relative abundance > 1%; Figure 1B).

**FIGURE 2:**
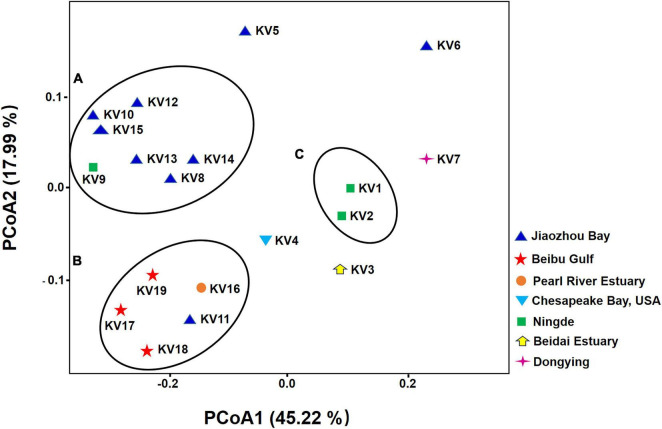
Principal coordinate analysis (PCoA) of bacterial community based on weighted-unifrac distances. The color of the symbols indicate the original isolation sources of samples.

The PCoA plot was performed to visualize similarity and difference of bacterial composition among different samples at the feature level (sequences clustered at 100% sequence similarity). Notably, the samples did not cluster according to their geographic origins (Figure 2). Six (KV8, KV10, KV12, KV13, KV14, KV15) of the 8 strains from Jiaozhou Bay (Shandong Province, China) and one (KV9) from Ningde (Fujian Province, China) formed cluster A. Two strains (KV1 and KV2) from Ningde (Fujian Province, China) formed cluster C. All the 3 strains from Beibu Gulf (Guangxi Province, China), one strain (KV16) from Pearl River Estuary (Guangdong Province, China), and one member (KV11) from Jiaozhou Bay (Shandong Province, China) formed cluster B (Figure 2). The remained 5 samples exhibited indiscernible affiliation with one another (Figure 2).

### Functional predictions of the whole bacterial microbiome of *Karlodinium veneficum*

**FIGURE 3:**
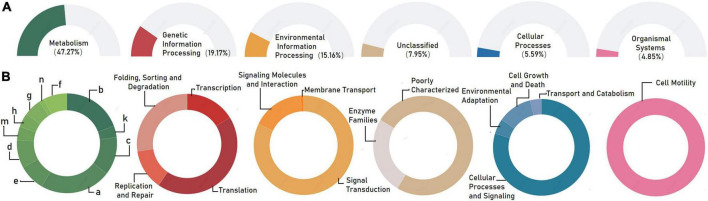
Functional prediction of the whole bacterial microbiome associated with *Karlodinium veneficum* in KEGG categories at level 1 **(A)** and level 2 **(B)**. Gene functions were predicted from 16S rRNA gene-based microbial compositions using the PICRUSt algorithm to make inferences from KEGG annotated databases. Relative signal intensity was normalized by the number of the genes for each indicated metabolic pathway. a: amino acid metabolism; b: carbohydrate metabolism; c: energy metabolism; d: metabolism of cofactors and vitamins; e: lipid metabolism; f: nucleotide metabolism; g: xenobiotics biodegradation and metabolism; h: glycan biosynthesis and metabolism; m: metabolism of terpenoids and polyketides; n: biosynthesis of other secondary metabolites.

To primarily disentangle the putative ecological roles played by the individual bacterial microbiomes in the associated phycospheres, the functional potentials of bacterial consortia were mapped to the reference pathways in the KEGG database. Generally, the whole bacterial microbiome showed high activity in the 3 terms representing major functions of metabolism (47.27%), genetic information processing (19.17%), and environmental information processing (15.16%) at the KEGG level 1, followed by terms of unclassified (7.95%), cellular processes (5.59%), and organismal systems (4.85%; Figure 3A). For the KEGG level 2, the terms membrane transport, amino acid metabolism, and carbohydrate metabolism exhibited relative high abundances to be the top three predominant functional modules (Figure 3B).

### Determination of the core (constitutive) microbiome of *Karlodinium veneficum*

**FIGURE 4:**
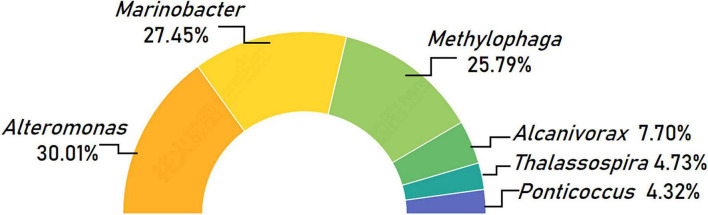
Taxonomic composition of core bacterial microbiome associated with *Karlodinium veneficum* across different strains at the genus level. Half circle diagram represents relative abundances of bacterial genera for the core bacterial microbiome.

The 6 genera, *Alteromonas*, *Marinobacter*, *Methylophaga*, *Alcanivorax*, *Thalassospira*, and *Ponticoccus*, were identified as the set of core bacterial microbiome living with *K. veneficum*, which were referred as “core genera” hereafter. These core genera account for 74.55% of the whole bacterial microbiome, in which *Alteromonas* was the most predominant, making up 30.01% of the core bacterial microbiome. The next two prevailing genera were *Marinobacter* (27.45%) and *Methylophaga* (25.79%; Figure 4). While *Alcanivorax* made up 5.74%, the genera *Thalassospira* and *Ponticoccus* both exceeded 4% in the core microbiome (Figure 4).

## Discussion

### Bacterial communities of high diversity associated with laboratory-cultured *Karlodinium veneficum* displayed heterogeneity in features composition among strains from different original sources (geographic origins)

Compared with the relatively high biodiversity observed in terrestrial plant microbiomes (e.g., rhizospheres typically harbor > 10^3^ bacterial taxa; Mendes et al., 2013 and references therein; Ling et al., 2022), some case-studies have reported less diversity in the bacterial communities of microalgae (Krohn-Molt et al., 2017). Especially for works employed laboratory-cultured microalgae, in which presumably well-adapted microbial communities have established during long-term maintenance (Buchan et al., 2014; Seymour et al., 2017). Generally, fewer than 30 bacterial isolates on a species level were affiliated with these microalgae-bacteria biofilms (Krohn-Molt et al., 2017 and the references therein). Here, the associated whole bacterial microbiome of dinoflagellate *K. veneficum* consisting of 1,417 features, with the number of recovered bacteria at the feature level per sample varying from 37 to 397 (mean = 76), reflecting high diversity and heterogeneity of bacterial flora harbored by laboratory cultures of *K. veneficum.* Metabolic inferences predicted broad and diverse functional repertoire of the whole bacterial communities in phycospheres, which still required further experimental validation. The PCoA analysis suggested that the geographic origins could influence the composition of the bacterial assemblages to some extent, as evident by the feature composition similarity shared by some strains from the same geographic origin. Yet strains with same and/or close sources could also displayed little or no affiliation with each other, indicating that the original sources was not necessarily the sole and critical determinant for their associated bacterial microbiome structures. We speculated that the excluded algal metabolites and chemical signals as well as the given cultivation conditions might shape and balance the associated bacterial communities of *K. veneficum* across different strains in our study.

### *Karlodinium veneficum* strains from different geographic origins harbor a core set of constitutive bacterial taxa in their phycospheres, which included three genera commonly reported as dinoflagellate-associated taxa

The whole bacterial microbiome of *K. veneficum* recovered in our study was generally congruent with the majority of previously characterized bacterial assemblages associated with laboratory cultures of dinoflagellates, in which α- and γ-proteobacteria are the predominant players (Li et al., 2019; Guo et al., 2020; Tarazona-Janampa et al., 2020; Deng et al., 2022; Wu et al., 2022; and the references therein). Despite the heterogeneity of feature composition, bacterial communities of strains from different geographic origins consisted a core set of constitutive taxa. Among them, the *Alteromonas* and *Marinobacter* (Alteromonadaceae) are the dominant phylotypes ubiquitously associated with a wide variety of dinoflagellates in both laboratory cultures and field samples (Mayali et al., 2011; Yang et al., 2015; Bolch et al., 2017; Park et al., 2018; Shin et al., 2018; Zhou et al., 2018; Hattenrath-Lehmann et al., 2019; Li et al., 2019; Cui et al., 2020; Guo et al., 2020; Tarazona-Janampa et al., 2020; Kang et al., 2021; Deng et al., 2022; Wu et al., 2022). Metabolically active *Marinobacter* may be essential for trace metal uptake of dinoflagellates via a beneficial interaction process well known as Iron-Carbon mutualism (Amin et al., 2009). While certain *Alteromonas* members exhibit algicidal/supportive effect on co-cultured phytoplankton depending on strain and relative abundance. They could inhibit phytoplankton growth by producing algicidal compounds or/and promote growth by removing hydrogen peroxide to relieve oxidative stress on phytoplankton (Aharonovich and Sher, 2016; Hennon et al., 2018). The genus *Methylophaga* is a unique group of aerobic, halophilic, non-methane-utilizing methylotrophs (Neufeld et al., 2007). Few documents have recorded their associations with dinoflagellates in field samples (Neufeld et al., 2008; Hattenrath-Lehmann et al., 2019), likely due to the carbon sources that methylotrophic bacteria depending on generally present at very low concentrations marine environments. Our recent study has revealed that *Methylophaga* could be the most significantly enriched bacterial group associated with laboratory-cultured dinoflagellates, relative to that associated with non-dinoflagellates (Deng et al., 2022). In the marine ecosystem, dinoflagellates are the most prolific producers of dimethylsulfoniopropionate (DMSP; Caruana and Malin, 2014). Case study on *K. veneficum* showed that its intracellular concentrations of DMSP could reach ∼11 mM (Caruana et al., 2012). DMSP could be subsequently degraded and released dimethylsulfide (DMS). The genus *Methylophaga* included the most majority of marine bacteria knowingly capable of using DMS as a sole carbon source (Boden et al., 2010). Plausibly, the tight association between *Methylophaga* and *K. veneficum* observed in our study support such notion.

### Highly specialized hydrocarbonoclastic bacteria stably living with *Karlodinium veneficum* implying that their association with dinoflagellates may be more common in nature than previously perceived

The hydrocarbonoclastic bacteria are prokaryotic hydrocarbon degraders capable of growing in pure culture using hydrocarbons as sole, or major source of carbon for growth (Prince et al., 2010; Thompson et al., 2017). Among them, obligate hydrocarbonoclastic bacteria (OHCB) are a unique group with narrow nutritional spectrum of hydrocarbons almost exclusively as a sole source of carbon and energy (Head et al., 2006; Yakimov et al., 2007). Of the 3 core genera reported here, *Alcanivorax*, *Thalassospira* and *Ponticoccus*, are among those hydrocarbon bacterial utilizers, while two of them (*Alcanivorax* and *Ponticoccus*) are recognized as obligate hydrocarbonoclastic taxa (Prince et al., 2010 and the references therein). *Alcanivorax* strains belonging to the γ-subclass of the Proteobacteria are merely able to grow on a highly restricted spectrum of substrates, predominantly linear and branched *n*-alkanes and their derivate, with carbon chain length ranging between 9 and 32 carbon molecules (Yakimov et al., 2007). Bacteria affiliated with the genus *Thalassospira* are marine α-proteobacteria that could degrade several polycyclic aromatic hydrocarbons (PAHs), including naphthalene, dibenzothiophene, phenanthrene and fluorene (Kodama et al., 2008). The γ-proteobacteria *Ponticoccus* are members that specialize in the PAHs degradation (Gutierrez et al., 2012).

In natural environment, despite the narrow nutritional requirement, hydrocarbonoclastic bacteria are nonetheless cosmopolitan and found throughout the global ocean. Interestingly, their global distribution appears to be solely confined to the marine environments, largely owing to oil-impacted seawater provide sources of carbon and energy to these hydrocarbon-degrading bacteria (see below). Petroleum is a natural, heterogeneous mixture of hydrocarbons, consisting mainly of alkanes with different chain lengths and branch points, cycloalkanes, mono-aromatic and polycyclic aromatic hydrocarbons (McGenity et al., 2012). Hundreds of millions of liters of petroleum enter the marine environment from both natural and anthropogenic sources every year (Head et al., 2006; McGenity et al., 2012). The ecological importance of hydrocarbonoclastic bacteria in the removal of hydrocarbon pollutants was evidenced in the reports documenting their strong enrichment in petrochemical input marine environments (Head et al., 2006; Yakimov et al., 2007 and references therein). In nature, they are usually present at low or undetectable levels before the polluting event and become predominant in petrochemical contamination seawater (Head et al., 2006; Yakimov et al., 2007). Until now, our knowledge about these bacteria (especially OHCB) is largely from few studies on seawater samples collected at petroleum-contaminated sites either by cultivation methods or sequencing surveys (Kodama et al., 2008; McGenity et al., 2012; Thompson et al., 2017). The ecology and physiology of hydrocarbon-degrading bacteria and particularly, their interactions with algae and other microbes during algal blooms remain to be explicitly explored.

Generally, the success of an algae-bacteria relationship has much to do with access, by the bacteria, to an available source of carbon and energy in the form of algal exudates (Bell and Mitchell, 1972) and reciprocal benefits to the phytoplankton through bacteria-mediated bioavailability (McGenity et al., 2012). Here the long-term (from >14 years to several months) and stable co-existence of 3 genera of hydrocarbon-degrading bacteria in the laboratory cultures of *K. veneficum* could be assumed to at least partly attribute to successful access of hydrocarbonoclastic bacteria to available source of hydrocarbon or hydrocarbon-like substrates (e.g., fatty acids, fatty alcohols, pristine, phytane, and PAHs). Dinoflagellates can be a biogenic source of hydrocarbons (Blumer et al., 1971; McKay et al., 1996; Shaw et al., 2010), and the excluded organic carbon could be subsequently utilized by bacteria such as OHCB. Moreover, either through biogenic synthesis or adsorption, the phytoplankton cell surface could accumulate PAHs (Andelman and Suess, 1970; Gutierrez et al., 2012, 2013; Thompson et al., 2017). Thus, the available hydrocarbon or hydrocarbon-like compounds in the phycosphere could play significant roles in supporting lives of the surrounding hydrocarbon-degrading bacteria, especially in the case for OHCB. However, it is not clear what the exact roles these prokaryotic hydrocarbon degraders played during their association with dinoflagellate hosts. Heretofore, only limited documents have reported the obligate and generalist hydrocarbonoclastic bacteria, either from laboratory cultures of dinoflagellates that have been maintained in cultivation for years, or through sequencing field samples of dinoflagellates freshly collected from places without recognized source of petrochemical input (Gutierrez et al., 2012, 2013; Shin et al., 2018; Guo et al., 2020; Tarazona-Janampa et al., 2020). The bacterial community associated with any non-axenic laboratory culture of phytoplankton is logically a close representative of the community that existed in the phycosperes of the cells in the field from which the culture strain was initially isolated and established (Jasti et al., 2005; Gutierrez et al., 2012, 2013). Hence, the observed stable hydrocarbonoclastic bacteria are very likely to be members of the microbiota associated with the 19 strains of *K. veneficum* at the time when they were originally established from different water sources. These findings collectively highlighted the occurrence of hydrocarbonoclastic bacteria, especially OHCB, with dinoflagellates may be more frequent in nature than what we have perceived. Our results also raised the possibility that the phycospheres of certain lineages of dinoflagellates (e.g., Kareniaceae) may represent a previously unrecognized niche attractive to bacterial taxa possessing specialized nutritional preference for hydrocarbons. This association brings important questions with respect to the roles that these prokaryotic hydrocarbon degraders played in their relationships with dinoflagellate hosts. Collectively, our results provided a stepwise gain of fundamental insights into the respective and interactive ecology of the key harmful *K. veneficum* and OHCB. The work also opened a window to further explore the ecological functions exerted by hydrocarbon degraders living with phytoplankton in natural seawaters.

## Data availability statement

The datasets presented in this study can be found in online repositories. The names of the repository/repositories and accession number(s) can be found in the article/Supplementary material.

## Author contributions

YD and KW performed the experiments, provided feedback on the experiments and results, and wrote the article with contributions of all authors. ZH maintained the algal cultures and prepared the samples. QH designed the research. YT designed the research and edited the manuscript. All authors read and approved the final manuscript.
