# Supplementary material for: Identification and implications of a core bacterial microbiome in 19 clonal cultures laboratory-reared for months to years of the cosmopolitan dinoflagellate Karlodinium veneficum
Source: Front Microbiol. 2022 Aug 4;13:967610. doi: 10.3389/fmicb.2022.967610 (PMC9416233; doi:10.3389/fmicb.2022.967610)
Supplement: Supplementary file 1 [file Data_Sheet_1.PDF]

Supplementary Figures

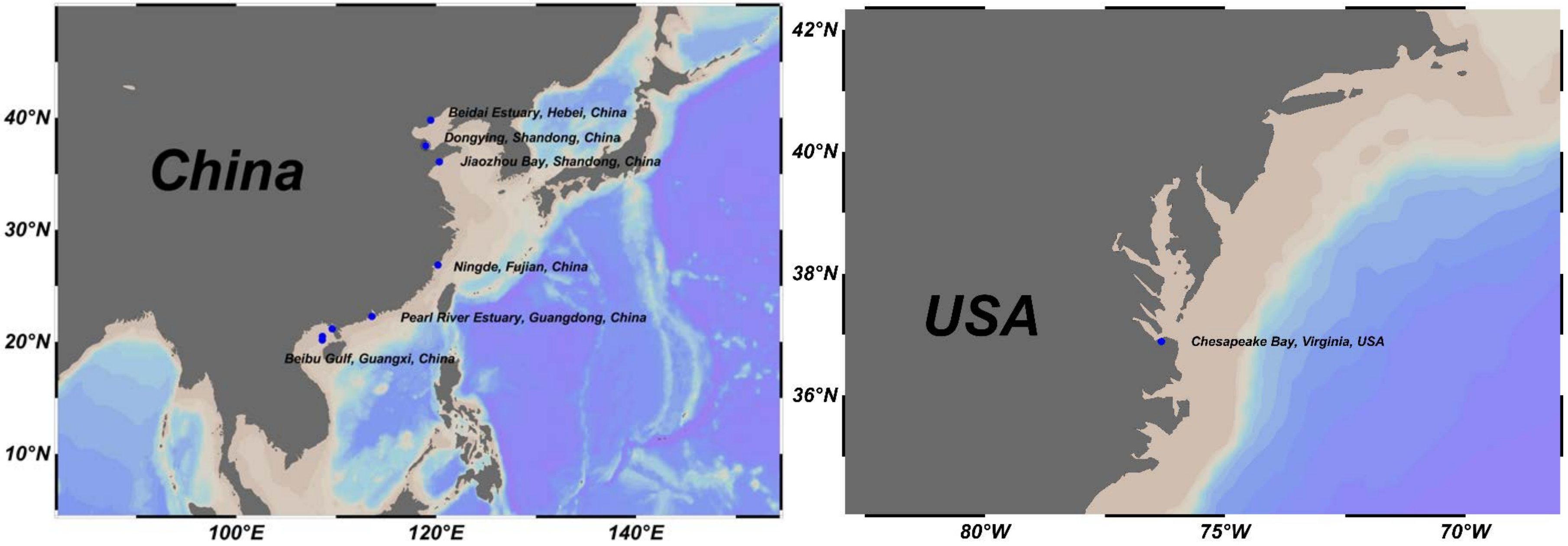

Supplementary Figure 1: The locations where the *Karlodinium veneficum* strains analyzed in this study were originally isolated (in blue filled circles).

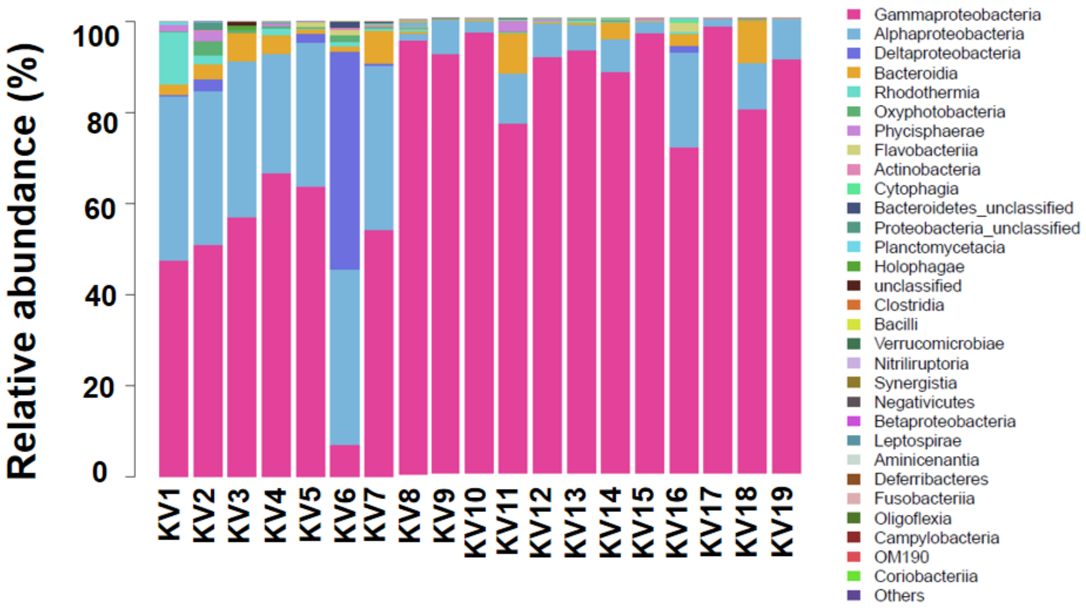

Supplementary Figure 2: Class-level relative abundance of bacterial communities (for the top 30 most abundant bacterial classes) in 19 *Karlodinium veneficum* samples.
